# Study on the Factors Affecting the Start-Up of Iron-Manganese Co-Oxide Filters for Ammonium and Manganese Removal from Groundwater

**DOI:** 10.3390/ijerph15091822

**Published:** 2018-08-23

**Authors:** Ya Cheng, Tinglin Huang, Lijie Cheng, Junbin Wu

**Affiliations:** 1 Key Laboratory of Northwest Resource, Environment and Ecology, Ministry of Education (MOE), Xi’an University of Architecture and Technology, Xi’an 710055, China; chengya.xauat@outlook.com (Y.C.); 15129883602@163.com (L.C.); junbingwu@foxmail.com (J.W.); 2 Shaanxi Key Laboratory of Environmental Engineering, Xi’an University of Architecture and Technology, Xi’an 710055, China

**Keywords:** iron-manganese co-oxide, filter, start-up, ammonium removal, manganese

## Abstract

The high concentration of ammonium (NH_4_^+^-N) and manganese (Mn^2+^) in underground water poses a major problem for drinking water treatment plants. Effective catalytic oxidative removal of NH_4_^+^-N and Mn^2+^ by iron-manganese co-oxide film (MeO_x_) filters was first developed by our group in a previous study. In this study, several identical pilot-scale filters were employed to optimize the start-up process for simultaneous removal of NH_4_^+^-N and Mn^2+^ from potable water supplies. Experiments were conducted to assess the influence of Mn^2+^ concentration, Fe^2+^ concentration, filtration rate and dosing time on the start-up period of the filter. Results demonstrated that the ability of the filter to remove completely 1.5 mg/L NH_4_^+^-N could be achieved on the sixth day at the soonest and the removal of Mn^2+^ could reach 1 mg/L by the 18th day. Filter R3 feeding with 1 mg/L Fe^2+^, 2 mg/L Mn^2+^ and 3.5 mg/L MnO_4_^−^ during the start-up period exhibited the optimum NH_4_^+^-N and Mn^2+^ removal effect. Short dosing time was not conducive to attaining full NH_4_^+^-N removal in filters, especially the activity of NO_2_^−^-N conversion to NO_3_^−^-N. The compositional analysis and element distribution analysis results demonstrated that there was an abundance of C, O, Mn, Mg, Fe, Ca and Si across the entire area of the surface of the filter media and the elemental distribution was homogeneous, which was different from the biofilter media. Knowledge-guided performance optimization of the active iron-manganese co-oxide could pave the way for its future technological use.

## 1. Introduction

Ammonium (NH_4_^+^-N) and manganese (Mn) contamination of groundwater is one of the problems faced in water treatment [1]. The presence of Mn in drinking water causes pipes to clog from the oxidation of Mn^2+^, which is precipitated as Mn^4+^ in the form of manganese oxide (MnO_2_), resulting in black colored water [2,3]. Importantly, Mn can cause neurotoxicity in humans. Additionally, in water supply plants, NH_4_^+^-N in raw water needs to be removed before the water is disinfected with chlorine, as it consumes significant amounts of chlorine and produces chloramines during the disinfection process [4]. The presence of NH_4_^+^-N in water systems also leads to oxygen depletion, toxicity to fish, eutrophication of surface waters, human nervous system damage and deterioration in the taste and odor of water [5]. The treatment of NH_4_^+^-N and Mn^2+^ to reduce the amount to below maximum concentration limits is a priority in drinking water treatment plants. Therefore, for some water supply plants, removal of NH_4_^+^-N and Mn^2+^ from groundwater is a daily requirement.

In order to solve this problem, various treatment technologies have been used to remove NH_4_^+^-N and Mn^2+^ from potable water, including biological and physicochemical methods [6,7,8,9,10]. Biofilters, as a biological treatment method, have been widely applied, with the advantages of simple processing technology, strong processing capacity and low investment cost [4,11,12]. However, the start-up period of the biological filter column usually takes months [13,14,15,16]. Hence, considerable effort has been made to improve designs for the efficient and economic biological removal of NH_4_^+^-N and Mn^2+^ [17]. Han et al. systematically investigated the effect of operation factors, such as hydraulic loading, air-to-water ratio and feed ammonium concentration, on simultaneous removal of NH_4_^+^-N and Mn^2+^ using a pilot-scale biological aerated filter. They demonstrated that distribution of ammonia oxidizing bacteria and manganese oxidizing bacteria could be a main factor affecting the system performance for simultaneous removal of NH_4_^+^-N and Mn^2+^ [18]. In order to shorten the start-up period of the biofilter for the removal of Fe^2+^, Mn^2+^ and NH_4_^+^-N simultaneously, Cai et al. introduced lab-scale biofilters with three different methods of inoculation. The start-up method that was most effective involved inoculation with nitrifying sludge first, followed by backwashing sludge obtained by backwashing the other matured Fe and Mn removal biofilters in the laboratory after complete NH_4_^+^-N removal. This method required 30 days to achieve complete removal of Mn^2+^ [19]. In addition, the sludge used in inoculation is a threat to the safety of drinking water treatment, because the sludge may contain metals or other potential pollutants [20].

Unlike biofilters, our group has employed physicochemical technology to start up filters which are used to enhance potable water quality by removing NH_4_^+^-N and Mn^2+^ [21,22]. Different from the above traditional biological NH_4_^+^-N and Mn^2+^ removal filter columns, the removal of NH_4_^+^-N was achieved by the chemical catalytic oxidation of iron-manganese co-oxide film coated on the filter media and not by biological degradation. The effective catalytic oxidation of NH_4_^+^-N by iron-manganese co-oxide film was already verified in our previous study [22]. The mineral structure of oxide film toward NH_4_^+^-N oxidation was studied systematically and the NH_4_^+^-N removal mechanisms by oxide film catalytic oxidation have been proposed [23].

The method used to shorten the start-up period is of considerable importance for the further application of this technology. In order to efficiently and economically remove NH_4_^+^-N and Mn^2+^, this study attempted to optimize the ripening periods of the removal filters to possibly help reduce the typically long ripening periods. This paper focuses on how to shorten the start-up period of the filter for the removal of NH_4_^+^-N and Mn^2+^, by starting up the pilot-scale filters with different feedwater. Pilot experiments were carried out to evaluate the influence of Mn^2+^ concentration, Fe^2+^ concentration, filtration rate and dosing time on the start-up of the filter.

## 2. Methods

### 2.1. Experimental Set-Up and Feed Water

**Figure 1:**
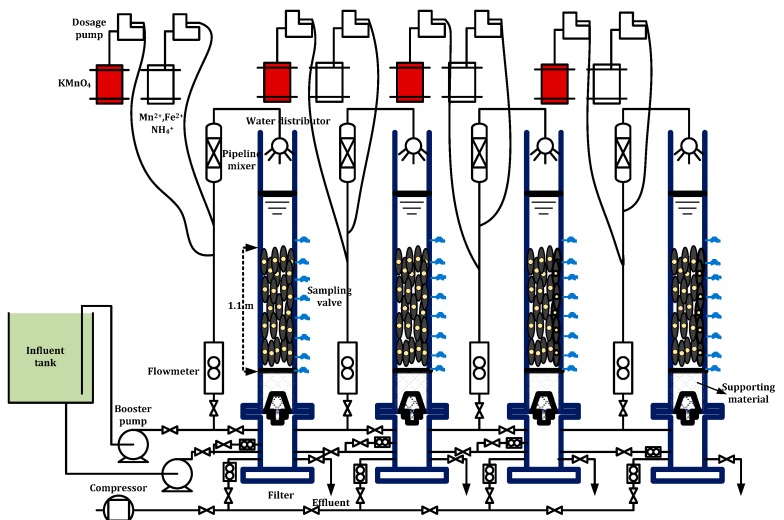
Schematic illustration of the pilot-scale filter system.

A gravity filter made of Plexiglass was adopted for the simultaneous NH_4_^+^-N and Mn^2+^ removal process. Each filter had an inner diameter of 100 mm and was filled with quartz sand of size fraction 0.75–1.2 mm, with a height of 1.10 m. Quartz sand was purchased from a local waterworks. Before the experiment, 1 mol/L HCl was used to soak the quartz sand for 1 day, then was rinsed off. In order to prevent the leakage of quartz sand, a layer of cobblestone was set below the sand as a supporting material with a height of 20 cm and a particle size of 8–15mm. The filter column was operated in down flow mode at a filtration efficiency of 4 m/h and backwashing process consisted of air backwash, air-water backwash and water backwash. In each step, the intensity of air and water backwash was 13–17 and 3–4 L/(s·m^2^), respectively. Eight sampling points (located at 0, 10, 22, 35, 55, 75, 95 and 115 cm from bottom to top) were attached along the height of the column to allow the sampling of water. A schematic diagram of the pilot scaled filter system is shown in Figure 1. The feed water of the filters used in the present study was groundwater obtained from a water source well in Xi’an, China. The compositions of the groundwater used in this study are provided in Table 1. Iron and manganese were nearly removed before using the groundwater for the experiment.

### 2.2. Start-Up Method for Filters

For starting up the filters, different start-up methods were applied to optimize the start-up procedure. Booster pumps and tubing were used to pump feed water through the system. The starting principle of the filters was as follows: mixing and dosing potassium permanganate (MnO_4_^−^), manganese chloride (Mn^2+^) and ferric chloride (Fe^2+^) with peristaltic pump; the formed oxides along with the raw water enter into the filter system; the oxides can be retained by the processes of filtration then forming a film of catalytically active metal oxide coated on the filter media, after prolonged filter runs. During the start-up period, MnO_4_^−^, Mn^2+^ and Fe^2+^ were mixed with a mole ratio of complete reaction, shown in Equations (1) and (2), to avoid excessive dosage of MnO_4_^−^, which would damage the development of the oxides film. During the operation period, in order to detect NH_4_^+^-N removal efficiency of the filter in time, NH_4_Cl was also dosed into the influent and the influent NH_4_^+^-N concentration was kept at 1.5 mg/L:

2MnO_4_^−^ + 3Mn^2+^ + 2H_2_O → 5MnO_2_ + 4H^+^ (1)

MnO_4_^−^ + 3Fe^2+^ + 4H^+^ → MnO_2_ + 3Fe^3+^ + 2H_2_O (2)

Several identical pilot-scale gravity filters were adopted in this study. Each filter system was operated continuously for four weeks. To investigate the effect of Mn^2+^ and Fe^2+^ concentration on the start-up of filter, Mn^2+^ and Fe^2+^ were manually dosed into the influent at a certain concentration and the filtration rate was maintained at 4 m/h. After 14 days’ operation, dosing MnO_4_^−^, Mn^2+^ and Fe^2+^ ceased. The ability to remove manganese was evaluated by varying the influent Mn^2+^ concentration of the filters on the 8th, 18th and 28th day. In order to study the effect of filtration rate on the start-up of the filters, the filtration rate increased from 4 to 6 m/h with other parameters kept constant. In addition, the effect of the dosing time (MnO_4_^−^, Mn^2+^ and Fe^2+^) on the start-up of the filters was also studied. Too high a Mn^2+^ and Fe^2+^ concentration could lead to too frequent backwashing. Therefore, in this study, the Fe^2+^ concentration was 0–2 mg/L, whereas the maximum Mn^2+^ concentration was set to 4 mg/L. The operating parameters of the filters (named R1, R2, R3, R4, R5, R6 and R7) in this study are presented in Table 2.

### 2.3. Analytical Methods

During the experimental runs, effluent samples were collected in polypropylene bottles. Manganese presented as Mn^2+^ was analyzed with the 1-(2-pyridylazo)-2-naphthol (PAN) method at an absorbance of 560 nm using a HACH DR 5000 instrument (Hach Company, Loveland, CO, USA). The levels of NH_4_^+^-N were determined spectrophotometrically with the Nesslerization method at an absorbance of 425 nm using the same HACH DR 5000 unit. Nitrate (NO_3_^−^-N) was analyzed by diazotization method also using the HACH DR 5000 and nitrite (NO_2_^−^-N) was also detected by spectrophotometry in accordance with standard methods. The dissolved oxygen (DO) and pH were determined by a portable instrument (HQ30d, Hach Company, Loveland, CO, USA).

A sample of 1.5 g of sand was taken from the top of each filtration column to carry out scanning electron microscope (SEM), Brunauer-Emmett-Teller (BET) and energy dispersive spectrometer (EDS) mapping testing. The morphology of the samples was observed using a field emission SEM (FEI Quanta 600F, Hillsboro, OR, USA). The surface and porous properties of the adsorbent were studied using nitrogen adsorption experiments realized with autosorb-1 (Quantachrome Instruments, Boynton Beach, FL, USA) at −196 °C with a heating rate of 10 K/min. The specific BET surface area and pore size of the adsorbent were evaluated according to the BET method. Multi-elemental EDS mapping was collected by an INCA IE350 x-ray spectrometer (Oxford Instruments, Abingdon, UK).

## 3. Results and Discussion

### 3.1. Effect of Fe^2+^ Concentrations on the Start-Up of the Filters

#### 3.1.1. The NH_4_^+^-N Removal Performance during the Start-Up Period

**Figure 2:**
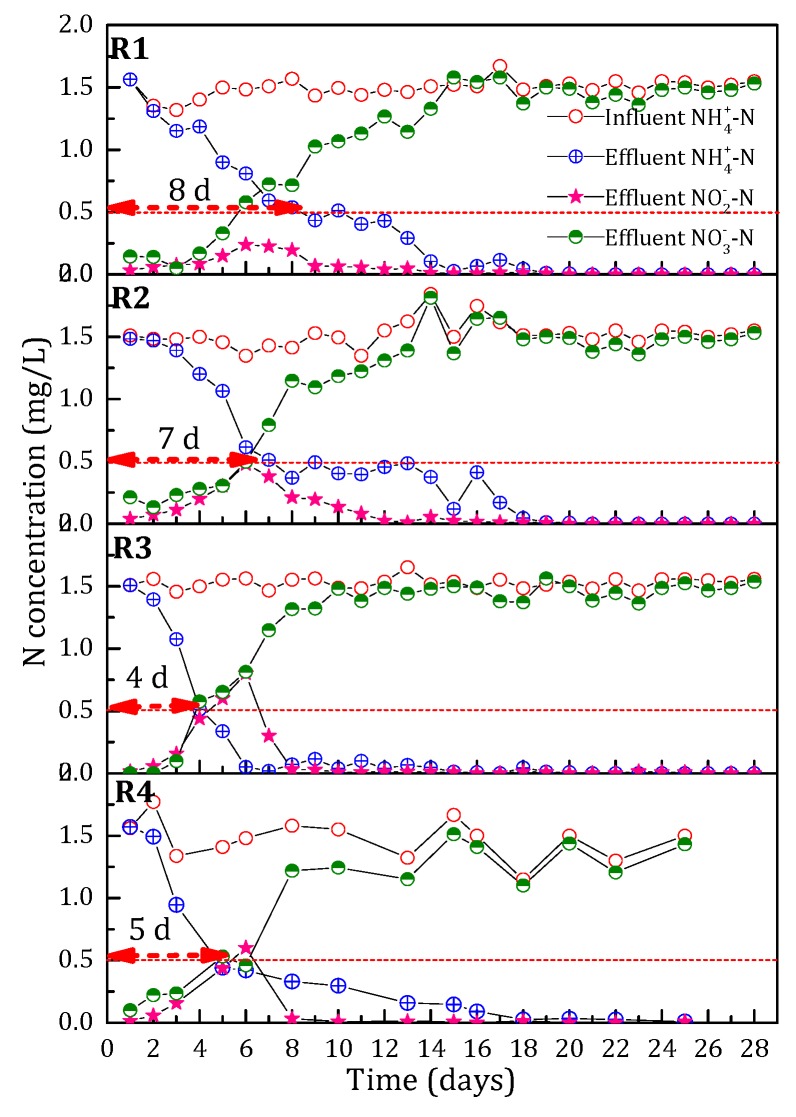
The effect of influent Fe^2+^ concentration on the variation of N (NH_4_^+^-N, NO_3_^−^-N and NO_2_^−^-N) concentration during the start-up period.

In this study, four identical pilot-scale filters were used to explore the effect of Fe^2+^ concentrations on the start-up of filter. The influent Fe^2+^ concentrations were 0 (filter R1), 0.5 (filter R2), 1 (filter R3) and 2.0 mg/L (filter R4). The experimental results are shown in Figure 2. During the first two days, the influent and effluent NH_4_^+^-N concentration of these four filters remained basically the same, which indicated that the adsorption capacity of virgin sand to NH_4_^+^-N could be ignored. From the third day, NH_4_^+^-N removal capacity of the filters increased gradually. At a nearly constant influent NH_4_^+^-N concentration of 1.5 mg/L, the effluent NH_4_^+^-N concentration of filters R1, R2, R3 and R4 could meet the drinking water quality standard in China (<0.5 mg/L) by the eighth, seventh, fourth and fifth day, respectively. With prolonging the running time, the influent NH_4_^+^-N could be eliminated at the 15th, 13th, 6th and 18th day for filters R1, R2, R3 and R4, respectively. The start-up period for the filters was much faster than that of the biofilters [15]. It should be noted that the big difference among the four filters was that the NH_4_^+^-N removal efficiency of filters R1, R2 and R4 from day 4 to 12 increased slower than that of filter R3.

During the first 10 days, the accumulation of NO_2_^−^-N was accompanied with rapidly increased NH_4_^+^-N removal capacity. Over time, NO_2_^−^-N was negatively correlated with the accumulation peak of NO_2_^−^-N. The accumulation time of NO_2_^−^-N: filter R1 (14 days) > filter R2 (12 days) > filter R4 (10 days) > filter R3 (8 days). The accumulation peak of NO_2_^−^-N in filter R3 with the shortest accumulation time was 0.8 mg/L. The accumulation peak of NO_2_^−^-N in filter R1 with the longest accumulation time was 0.24 mg/L. Jiang et al. reported that the accumulation of NO_2_^−^-N in biofilters was related to the level of DO condition [24]. However, in this study, the influent DO of all the filters was almost the same, which could meet the oxygen demand of NH_4_^+^-N removal. So it could be inferred that the accumulation of NO_2_^−^-N was due to the lack of NO_2_^−^-N catalytic oxidation activity of the filter media.

#### 3.1.2. Mn^2+^ Removal Performance during the Start-Up Period

**Figure 3:**
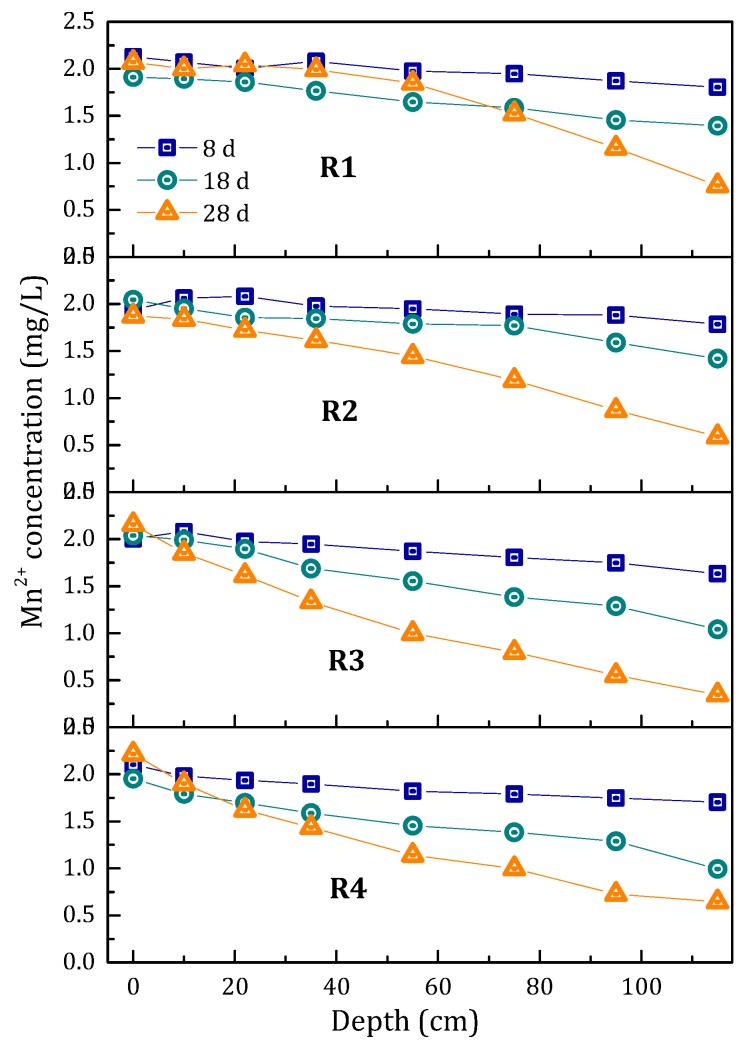
The effect of influent Fe^2+^ concentration on the variation of Mn^2+^ concentration along the depth of the filters at different running time.

During the start-up period, MnO_4_^−^, Mn^2+^ and Fe^2+^ reacted completely and Mn^2+^ and Fe^2+^ were hardly detectable in the influent water. In order to investigate the variation in manganese removal capacity of the filters with running time, the experiments were conducted on the 8th, 18th and 28th day. The concentration of Mn^2+^ in the influent was kept at 2 mg/L. The variation of Mn^2+^ concentration along the depth of the filters is shown in Figure 3. The removal capacity of the filter column along the depth of the filter gradually increased.

**Figure 4:**
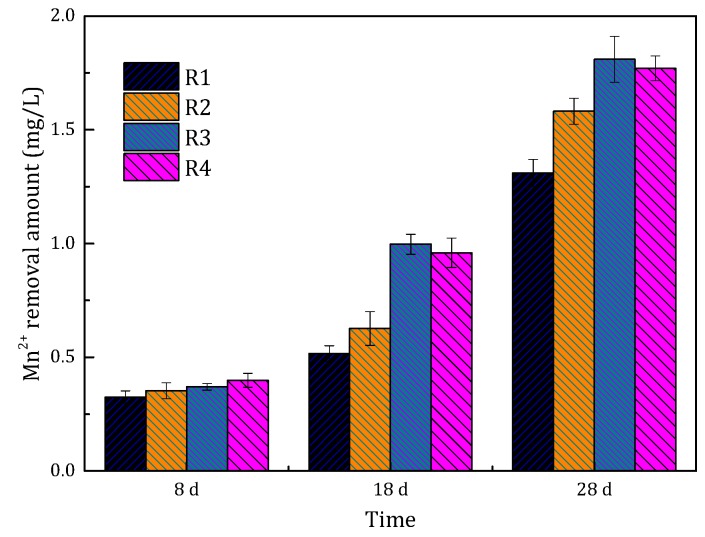
The comparison of the Mn^2+^ removal of the filters (R1, R2, R3 and R4) at different running time.

The Mn^2+^ removal of these four filters is presented in Figure 4. The Mn^2+^ removal amount also increased with running time. At the eighth day, the Mn^2+^ removal amount was nearly the same for all filters. However, the Mn^2^^+^ removal capability of the filters became different afterward, which could be attributed to the different catalytic activities of the filter media. The Mn^2+^ removal capacity of filters R3 and R4 was higher than that of the other two filters. Considering the dosing concentration of filter R3 was lower than that of filter R4, we concluded that a higher Fe^2+^ concentration of 1 mg/L produces better results during the start-up period.

#### 3.1.3. Variation in pH and DO during the Start-up Period

**Figure 5:**
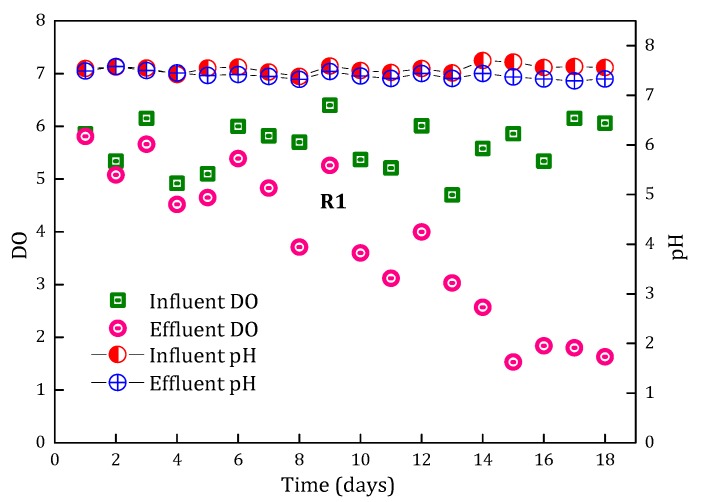
The variation of pH and dissolved oxygen (DO) in filter R1 during the start-up period.

Filter R1 was selected to study the variation in pH and DO with the running time during the operation period, as shown in Figure 5. Theoretically, hydrogen ions (H^+^) would be released when NH_4_^+^-N oxidizes to NO_2_^−^-N or NO_3_^−^-N:

NH_4_^+^ + 1.5O_2_ → NO_2_^−^ + H_2_O + 2H^+^ (3)

NH_4_^+^ + 2O_2_ → NO_3_^−^ + H_2_O + 2H^+^ (4)

However, compared with the influent pH, the effluent pH did not change much. This was mainly attributed to the buffering effect of the groundwater with high alkalinity (Table 1). The influent DO remained at 5–7 mg/L. The effluent DO decreased gradually along with the running time, which was in accordance with the NH_4_^+^-N removal performance (Figure 2). Especially in the first 15 days, the effluent NH_4_^+^-N and DO concentration decreased synchronously, but it’s worth noting that the DO consumption (5 mg/L) for NH_4_^+^-N (1.5 mg/L) removal was less than the theoretical DO consumption (6.8 mg/L) according to Equation (4), which should be caused by the atmospheric reoxygenation during sampling and testing process. Nonetheless, the NH_4_^+^-N removal capacity of the filters during the start-up period could be evaluated by detecting the change in DO in the influent and effluent.

### 3.2. Effect of Mn^2+^ Concentration on the Start-Up of the Filters

#### 3.2.1. NH_4_^+^-N Removal Performance during the Start-Up Period

**Figure 6:**
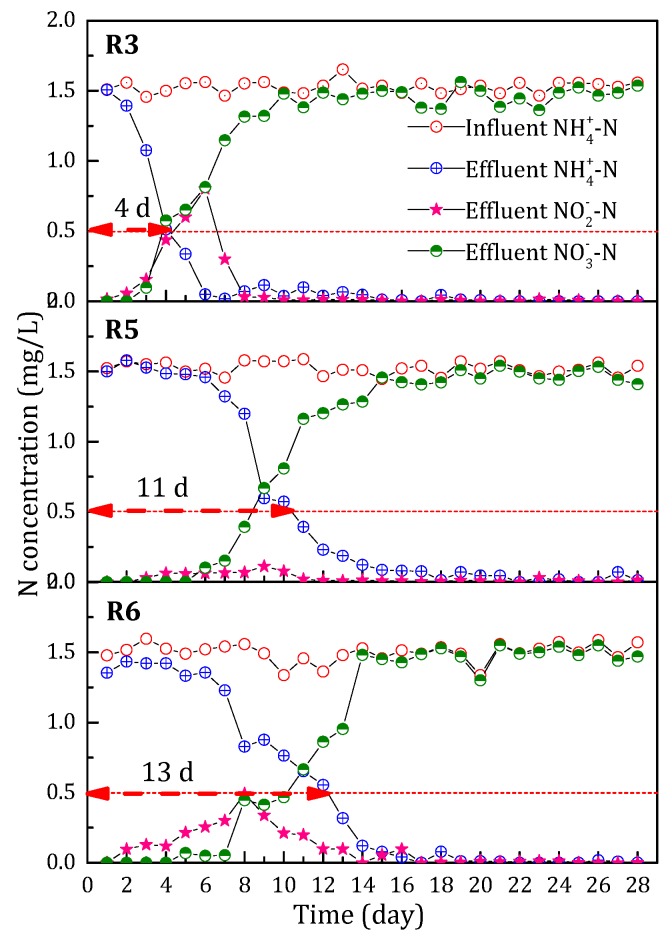
The effect of influent Mn concentration and filtration rate on the variation of N (NH_4_^+^-N, NO_3_^−^-N and NO_2_^−^-N) concentration during the start-up period.

As known from the above experimental results, the start-up of filter R3 performed the best. Therefore, in order to further explore the effect of Mn^2+^ concentration on the start-up of the filters for NH_4_^+^-N and Mn^2+^ removal, a filter named R5 was initiated. During the start-up period, the influent Fe^2+^ and NH_4_^+^-N concentration of filter R5 was the same as that of R3. The difference was that the influent Mn^2+^ concentration increased from 2 to 4 mg/L. The comparison of NH_4_^+^-N removal performance during the start-up period between filter R3 and R5 is shown in Figure 6. Filter R3 with low influent Mn concentration (2 mg/L) demonstrated better NH_4_^+^-N removal performance than that of filter R5 with high influent Mn^2+^ concentration (4 mg/L). The time at which capacity of NH_4_^+^-N catalytic oxidation started extended from the first day (filter R3) to the sixth day (filter R5). The effluent NH_4_^+^-N concentration of filter R3 met the drinking water quality standard in China (<0.5 mg/L) on the fourth day, but the time for filter R5 to obtain the same level of effluent NH_4_^+^-N concentration required 11 days. Hence, too high a Mn^2+^ concentration was not beneficial to the formation of NH_4_^+^-N removal capability of the filters. In addition, we also found that little accumulation of NO_2_^−^-N appeared in filter R5. This implied that high Mn^2+^ concentrations were beneficial to the transformation of NO_2_^−^-N.

#### 3.2.2. Mn^2+^ Removal Performance during the Start-Up Period

**Figure 7:**
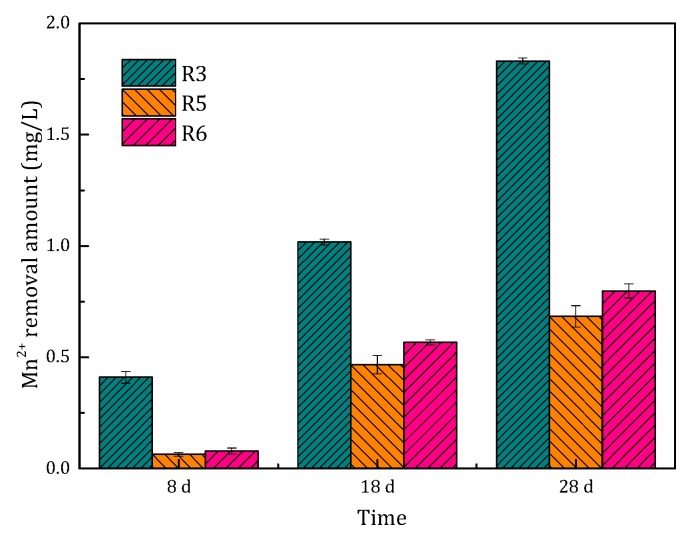
Comparison of the Mn^2+^ removal amount of the filters (R3, R5 and R6) at different running times.

The comparison of the Mn^2+^ removal amount in filters R3 and R5 is shown in Figure 7. The Mn^2+^ removal ability of filters R3 and R5 increased with the running time. The Mn^2+^ removal performance of filter R3 was much better than that of the filter R5. The Mn^2+^ removal ability of filter R5 on the 28th day was even lower than that of filter R3 on the 18th day. During the actual operation of the filters, we found that the frequency of backwashing may affect the Mn^2+^ removal performance of the filters. Backwashing was carried out every day in filter R5, which was more frequent than for filter R3 (every two days). The higher the Mn^2+^ concentration, the more frequent the backwashing. Frequent backwashing in the filter scoured the oxides coated on the filter media, which resulted in unstable coating of the oxides. Hence, the ability to remove Mn^2+^ in filter R5 was weak.

### 3.3. Effect of Filtration Rate on the Start-Up of the Filters

#### 3.3.1. NH_4_^+^-N Removal Performance during the Start-Up Period

In order to further explore the effect of filtration rate on the start-up of the filters for NH_4_^+^-N removal, a filter named R6 was adopted. During the start-up period, the influent MnO_4_^−^, Fe^2+^, Mn^2+^ and NH_4_^+^-N concentrations of the R6 filter were the same as that of R3. The only difference was that filtration rate was changed from 4 m/h to 6 m/h. As shown in Figure 6, the increase of filtration rate had little effect on the removal capacity of NH_4_^+^-N, but it resulted in the obvious accumulation of NO_2_^−^-N. The results indicated that the transformation from NO_2_^−^-N to NO_3_^−^-N required longer reaction time than from NH_4_^+^-N to NO_2_^−^-N.

#### 3.3.2. The Mn^2+^ Removal Performance during the Start-Up Period

The comparison of Mn^2+^ removal amount between filter R5 and R6 is shown in Figure 7. The Mn^2+^ removal ability of filter R5 and R6 gradually increased with the running time and was basically the same. However, it is worth noting that the Mn^2+^ removal ability of filters R5 and R6 was significantly worse than that of filter R3. This phenomenon was closely related to the frequent backwashing as discussed in Section 3.2.2. During the start-up period, the increase in the filtration rate led to the increase in the influent loads of the filter, similar to the high influent Mn^2+^ concentration. Backwashing was carried out every 12 h in filter R5. Therefore, in this study, the optimal filtration rate was 4 m/h during the start-up period.

### 3.4. Effect of Dosing Time on the NH_4_^+^-N Removal Performance 

The duration of dosing time determines whether a start-up method is economical. For all filters dosage of MnO_4_^−^, Fe^2+^ and Mn^2+^ was stopped at the 14th day. At this point, the effluent NH_4_^+^-N concentration of the filters could basically satisfy the limited stated in the drinking water quality standard in China and the concentration of NO_2_^−^-N was also very low. Therefore, in order to further shorten the start-up period and reduce the cost, dosing time was optimized.

**Figure 8:**
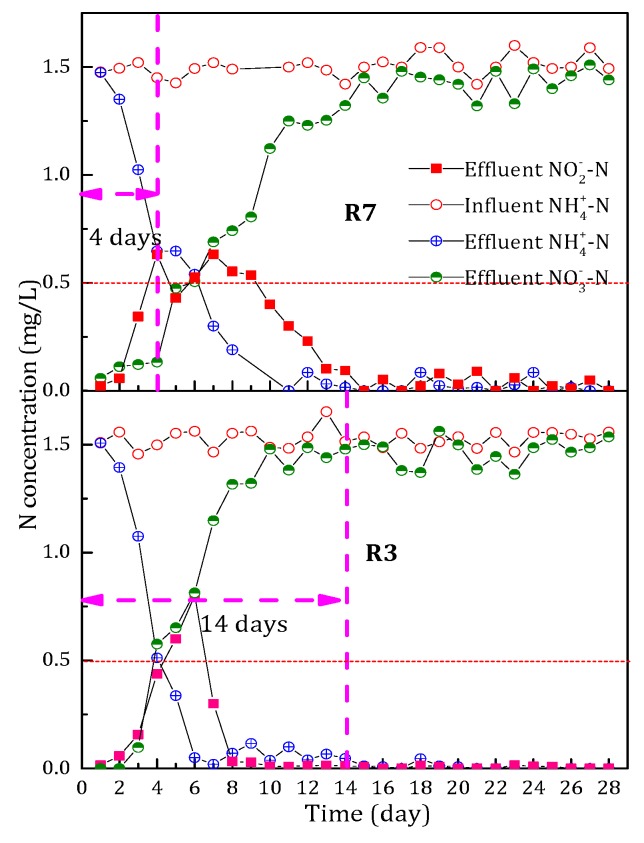
The effect of dosing time on the variation of N (NH_4_^+^-N, NO_3_^−^-N and NO_2_^−^-N) concentration during the start-up period.

A filter named R7 was initiated with the same start-up method as filter R3. However, the dosing time decreased from 14 days to four days. The comparison of NH_4_^+^-N removal performance is shown in Figure 8.

On the fourth day, the ability of NH_4_^+^-N removal of filter R7 was established. At this time, dosing MnO_4_^−^, Fe^2+^ and Mn^2+^ was stopped. After then, we found that the rate of NH_4_^+^-N removal decreased in filter R7. The time for NH_4_^+^-N removal completely extended from day 7 to 11. Meanwhile, the accumulation of NO_2_^−^-N became more obvious. The required time extended from 8 days to 15 days when the effluent was completely free of NO_2_^−^-N. Therefore, we concluded that the time when adding MnO_4_^−^, Fe^2+^ and Mn^2+^ was too short and not conducive to the full formation of the activity of NH_4_^+^-N removal in filters, especially the activity of NO_2_^−^-N conversion to NO_3_^−^-N.

### 3.5. Characterization Studies

#### 3.5.1. Morphological Analysis

**Figure 9:**
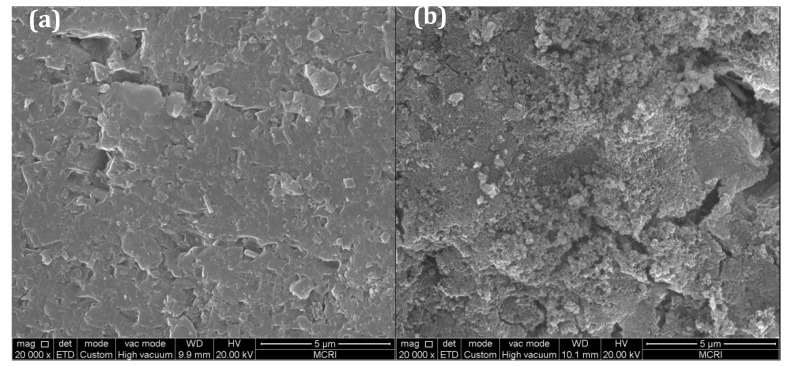
Scanning electronic microscope images of (**a**) virgin sand and (**b**) the filter media collected from filter R3 on the 12th day.

The surfaces of the virgin sand and the filter media in filter R3 collected on the 12th day were characterized by SEM, as shown in Figure 9. From Figure 9a, the surface of the virgin sand was smooth. After 12 days’ running, the surface of the sand was coated with many oxides, which were formed by the accumulation of small particles, as shown in Figure 9b. Specific surface areas of these samples were further determined using the BET method. The results showed that the specific surface area increased from 0.106 m^2^/g (virgin sand) to 3.920 m^2^/g (filter media collected on 12th day). Cao et al. reported that the increased specific surface area provided more active sites for NH_4_^+^-N catalytic oxidation, which played a decisive role in the catalytic oxidation process of NH_4_^+^-N [25].

#### 3.5.2. Composition Analysis

The surface element composition of the virgin sand and the filter media collected on the 12th day was analyzed by EDS. As shown in Table 3, virgin sand mainly contained two elements: silicon (Si) and oxygen (O). After 12 days’ operation, the oxides formed on the surface of the sand contained C, Mn, Fe, Mg, Ca, Si and O elements, which was consistent with our previous study [26]. Due to the minimal change in the total organic content (TOC) along the depth of the filter (data not shown), we inferred that the C mainly arose from the alkalinity of the water and existed in the form of calcium carbonate and manganese carbonate, which was confirmed in previous studies [27].

#### 3.5.3. Element Distribution Analysis

**Figure 10:**
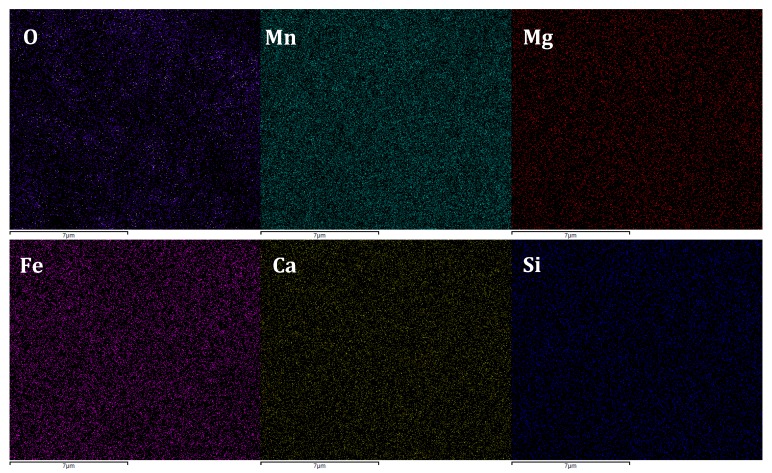
Multi-element energy dispersive spectrometry (EDS) mapping images of the filter media collected on the 20th day in filter R3.

Multi-elemental EDS mapping images of the surface of the filter media are shown in Figure 10. Apparently, there was an abundance of O, Mn, Mg, Fe, Ca and Si. And the elemental distribution was homogeneous across the entire area of the surface of the filter media. According to the literature, the characteristic elemental distribution on the surface of filter media in this study was much different that of biological filter media. Heterogeneous distribution of Fe, Mn and C was observed on the surface of an “aged” biofilter media [28]. Alain et al. reported that the origin of the coating was a possible cause for chemical heterogeneity in surface coatings [29].

### 3.6. Comparison with Other Studies

Based on our previous research [21], the start-up period of the filter column was further optimized in this study. The method for oxidation of Mn^2+^ and Fe^2+^ by MnO_4_^−^ during the start-up period could guarantee that effluent NH_4_^+^-N would meet the drinking water standard by the fourth day, when the influent NH_4_^+^-N was 1.5 mg/L. On the 18th day, the removal amount of Mn^2+^ reached 1 mg/L, which would meet most of the drinking water treatment requirements. A comparison of the start-up period and the removal performance of NH_4_^+^-N and Mn^2+^ with other studies is shown in Table 4. Compared with other start-up methods, the method in our study has obvious advantages.

## 4. Conclusions

In the present study, an optimization method for the start-up of a NH_4_^+^-N and Mn^2+^ removal filter for drinking water treatment was proposed. The chemical oxidation method effectively shortened the start-up period of the filters. The operational factors of the influent Mn^2+^ concentration, Fe^2+^ concentration, filtration rate and dosing time were optimized. The optimum conditions were found to be 1.5 mg/L NH_4_^+^-N, 2 mg/L Mn^2+^, 1.5 mg/L Fe^2+^, a 4 m/h filtration rate and the duration of dosing time was 14 days. During the start-up period, the specific surface area of the filter media increased along with prolonging the running time, which provided more active sites for NH_4_^+^-N catalytic oxidation. During the start-up period, frequent backwashing was not favorable to the formation of the NH_4_^+^-N and Mn^2+^ removal ability of the filters. The start-up periods of all filters in this study were much faster than those of biofilters. This study has shown that the use of an iron-manganese co-oxide filter could be an effective system for catalytic oxidation to remove NH_4_^+^-N and Mn^2+^ from drinking water.

## Figures and Tables

**Table 1:** Chemical composition of the feed water.

Parameters	Unit	Value
Cl^−^	mg/L	60–65
SO_4_^2^^−^	mg/L	95–110
Ca^2+^	mg/L	35–40
Mg^2+^	mg/L	23–25
Temperature	°C	15–18
pH	-	7.5–7.8
Alkalinity (CaCO_3_)	mg/L	200–250
Fe	mg/L	0–0.13
Mn	mg/L	0–0.08
NH_4_^+^-N	mg/L	0–0.2
NO_3_^−^-N	mg/L	0–0.40
NO_2_^−^-N	mg/L	0–0.003
TOC	mg/L	1–3.5
Na^+^	mg/L	80–90
DO	mg/L	2.0–4.0

TOC: Total organic content; DO: Dissolved oxygen.

**Table 2:** The operating parameters of all the filters during operation period (start-up period and normal operation period).

Name	Start-Up Period Normal							Operation Period		
	c(Mn^2+^) (mg/L)	c(Fe^2+^) (mg/L)	c(MnO_4_^−^) (mg/L)	c(NH_4_^+^-N) (mg/L)	*v* (m/h)	Backwashing Frequency	T * (day)	c(NH_4_^+^-N) (mg/L)	*v* (m/h)	Backwashing Frequency
R1	2	0	2.8	1.5	4	2 days	14	1.5	6	2 days
R2	2	0.5	3.2	1.5	4	2 days	14	1.5	6	2 days
R3	2	1.0	3.5	1.5	4	2 days	14	1.5	6	2 days
R4	2	2.0	4.2	1.5	4	2 days	14	1.5	6	2 days
R5	4	1.0	6.4	1.5	4	1 days	14	1.5	6	2 days
R6	4	1.0	6.4	1.5	6	12 hours	14	1.5	6	2 days
R7	2	1.0	3.5	1.5	4	2 days	4	1.5	6	2 days

T *: The time of dosing MnO_4_^−^, Mn^2+^ and Fe^2+^ (Start-up period).

**Table 3:** The elemental composition of the virgin sand and the filter media.

Element.	C	O	Na	Mg	Si	Ca	Mn	Fe
Virgin sand (%)		65.38			34.62			
Filter media (%)	20.74	52.29	0.91	0.70	10.62	1.68	8.46	4.60

**Table 4:** The comparison of the start-up period of NH_4_^+^-N and Mn^2+^ with other studies.

Treatment System	Media Types	Temperature	DO (mg/L)	pH	Backwashing	c(NH_4_^+^-N) (mg/L)		Period (day)	c(Mn) (mg/L)		Period (Day)	Reference
						Influent	Effluent		Influent	Effluent		
Pilot-scale filter	Quartz sand	15–18	5–7	7.5–7.8	2 day	1.5	<0.1	6	1.0	<0.1	18	This study
Pilot-scale filter	Quartz sand	6.6–22	6.5–7.0	7.9–8.1	-	1.39 ± 0.10	0.28	19	0.99 ± 0.12	<0.1	26	[22]
Pilot-scale biofilter	Mn Sand	8	8	-	2 day	1.4	<0.1	31	1.2	<0.05	51	[15]
Pilot Mn removal filter	Quartz sand	10.5–12.5	8–9.5	7.5–7.9	2 weeks	-		-	0.1–0.15	0	25	[30]
Bench-scale biofilter	Sand	-	reducing conditions	6.5	-	-		-	0.1–0.3	<0.05	42	[12]
Lab-scale biofilters	Quartz sand	18–22	7.8–8.5	7.5–7.8	-	1.2	<0.1	13	0.8	<0.05	30	[19]
Lab-scale biofilters	Sand	>20	7.5–8	7.7	weekly	-		-	0.5	0	90	[2]

DO: Dissolved oxygen.
